# Spatial-temporal heterogeneity in malaria receptivity is best estimated by vector biting rates in areas nearing elimination

**DOI:** 10.1186/s13071-018-3201-1

**Published:** 2018-11-27

**Authors:** Thomas R. Burkot, Hugo Bugoro, Allan Apairamo, Robert D. Cooper, Diego F. Echeverry, Danyal Odabasi, Nigel W. Beebe, Victoria Makuru, Honglin Xiao, Jenna R. Davidson, Nicholas A. Deason, Hedrick Reuben, James W. Kazura, Frank H. Collins, Neil F. Lobo, Tanya L. Russell

**Affiliations:** 1 James Cook University, Australian Institute of Tropical Health and Medicine, Cairns, QLD 4870 Australia; 2 National Vector Borne Disease Control Programme, Ministry of Health and Medical Services, Honiara, Solomon Islands; 3 Research Department, Solomon Islands National University, Honiara, Solomon Islands; 4 Australian Army Malaria Institute, Gallipoli Barracks, Enoggera, 4052 Australia; 5 Department of Biological Sciences, Eck Institute for Global Health, University of Notre Dame, Notre Dame, IN 46556 USA; 6 Swiss Tropical and Public Health Institute, Basel, Switzerland; 7 University of Basel, Basel, Switzerland; 8 University of Queensland, School of Biological Sciences, QLD, St. Lucia, 4068 Australia; 9 CSIRO, Dutton Park, Brisbane, QLD 4102 Australia; 10 Western Province Malaria Control, Gizo, Western Province Solomon Islands; 11 Center for Global Health & Diseases, Case Western Reserve University School of Medicine, Cleveland, Ohio 44106–4983 USA

**Keywords:** *Anopheles farauti*, *Anopheles hinesorum*, Malaria, Receptivity, Elimination, Solomon Islands

## Abstract

**Background:**

Decisions on when vector control can be withdrawn after malaria is eliminated depend on the receptivity or potential of an area to support vector populations. To guide malaria control and elimination programmes, the potential of biting rates, sporozoite rates, entomological inoculation rates and parity rates to estimate malaria receptivity and transmission were compared within and among geographically localised villages of active transmission in the Western Province of the Solomon Islands.

**Results:**

Malaria transmission and transmission potential was heterogeneous in both time and space both among and within villages as defined by anopheline species composition and biting densities. Biting rates during the peak biting period (from 18:00 to 00:00 h) of the primary vector, *Anopheles farauti*, ranged from less than 0.3 bites per person per half night in low receptivity villages to 26 bites per person in highly receptive villages. Within villages, sites with high anopheline biting rates were significantly clustered. Sporozoite rates provided evidence for continued transmission of *Plasmodium falciparum*, *P. vivax* and *P. ovale* by *An. farauti* and for incriminating *An. hinesorum*, as a minor vector, but were unreliable as indicators of transmission intensity.

**Conclusions:**

In the low transmission area studied, sporozoite, entomological inoculation and parity rates could not be measured with the precision required to provide guidance to malaria programmes. Receptivity and potential transmission risk may be most reliably estimated by the vector biting rate. These results support the meaningful design of operational research programmes to ensure that resources are focused on providing information that can be utilised by malaria control programmes to best understand both transmission, transmission risk and receptivity across different areas.

## Background

Globally, malaria transmission has fallen significantly with 68% of the reduction in *Plasmodium falciparum* in Africa attributed to the use of long-lasting insecticide-treated nets (LLINs) [1]. Recently, the World Health Assembly endorsed the Global Technical Strategy for Malaria Control and Elimination (GTS) [2]. The GTS recommends universal access to vector control with LLINs or indoor residual spraying (IRS) to all people at-risk of malaria. Larval source management (including larviciding, insect growth regulators and environmental management) is also recommended in the GTS as a supplemental control measure where larval habitats are few in number, fixed in location and easily accessible. Maintenance of universal access to LLINs or IRS after elimination is recommended in areas both receptive and vulnerable to malaria [2]. Withdrawal of universal access to LLINs or IRS after malaria is eliminated will depend on the risk of resumption of transmission if the malaria parasite is re-introduced, a function of the movement of infected people or mosquitoes (e.g. vulnerability) and vector receptivity (e.g. an environment inherently capable of supporting significant vector populations) [3]. Receptivity is not static and can change with urbanization, alterations in land use patterns and implementation of interventions that permanently reduce the vectorial capacity. Hence, there is a need to better understand how to quantify receptivity to guide malaria elimination programmes.

Although the Solomon Islands has achieved significant reductions in malaria transmission across the past decade, the number of cases has increased since 2015 and the annual parasite incidence was 83.4 cases/1000 population in 2017 (Solomon Islands Ministry of Health and Medical Services, unpublished data). In Western Province, the annual parasite incidence was only 7/1000 population in 2015 and foci of transmission emerged. Concurrently, the malaria parasite ratio changed from predominantly *P. falciparum* to *P. vivax*. Stratifying areas by receptivity will be important for targeting resources to where they are required to maintain malaria elimination as well as to respond rapidly to outbreaks [4, 5]. For entomological monitoring of adult anophelines in foci investigations, determining the species composition, receptivity and insecticide resistance are the highest priorities with moderate emphasis on determining the human biting rate, biting time and location [6].

Malaria in the Solomon Islands is transmitted almost exclusively by *An. farauti*. Following exposure to DDT applied in IRS during the original Malaria Elimination Programme of the 1970s, *An. farauti* shifted its biting profile from all night with both indoor and outdoor biting to predominantly biting early in the evening and outdoors [7–9]. While this behavioural change enables this mosquito to minimize contact with the WHO recommended interventions (LLINs and IRS) that are applied inside houses, these tools still retain efficacy. This is due to *An. farauti* having a short feeding cycle and individual mosquitoes must complete 5–6 feeding cycles to live long enough to complete the extrinsic incubation period and be infectious. During this time, they are likely to feed indoors at least once, and so, LLINs and IRS can potentially kill a significant proportion of the population [10]. The impacts of LLINs and IRS on vectorial capacity are temporary, and premature withdrawal of these interventions, in the absence of other interventions to permanently reduce vectorial capacity, will leave such areas susceptible to malaria resurgence [3, 11–13].

In this context, the utility of different entomological indicators to estimate malaria receptivity, transmission and transmission potential across time and space were directly compared, being biting rates, survivorship (by parity dissections), sporozoite rates and entomological inoculation rates of all human-biting anophelines. The comparison was made across 11 villages that spanned a malaria foci in the Western Province of the Solomon Islands.

## Methods

### Study sites

**Fig. 1:**
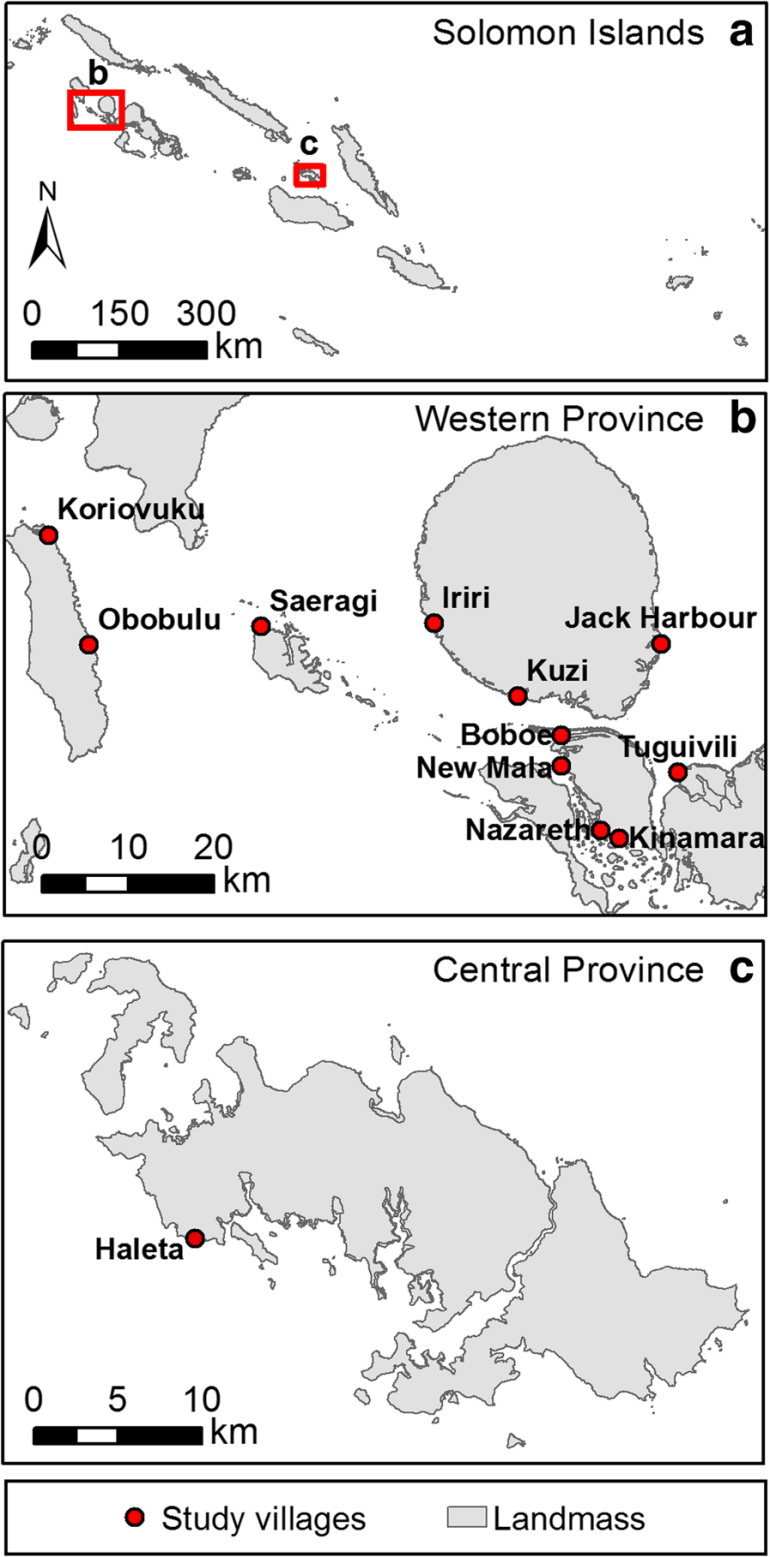
Map of (**a**) the Solomon Islands showing (**b**) the 11 study villages in Western Province (-8°0'S, 157°0'E) and (**c**) Central Province (-9°0'S, 159°45'E)

The study was conducted in 11 coastal villages located on the volcanic, mountainous, rain-forested islands of Ghizo, Kohinggo, Kolombangara, New Georgia and Ranonnga Islands of Western Province of the Solomon Islands (-8°0'S, 157°0'E) [14]; as well as in Haleta Village on Ngella Sule Island in Central Province (-9°0'S, 159°45'E) (Fig. 1) [10, 14–16]. Prior to commencing the entomological survey, the malaria prevalence across the study villages in Western Province was estimated to be 2.1% (measured by PCR in 2013, unpublished data). The area is classified by the Solomon Islands Government as a “near elimination” province. In contrast, Central Province had a malaria prevalence of 13.4% (as measured by PCR in 2012) [17]. Spatial analyses of malaria prevalence in humans from a cross-sectional survey conducted in 2013 identified a significant foci of infection that encompassed the villages of Jack Harbour and Tugivili, but not the villages of Nazareth, Kinamara or Saeragi with New Mala situated on the periphery of the foci (unpublished data).

Western Province encompasses ≈5000 km^2^ with a population of 76,649 in 13,762 households [18]. The climate of the region is hot and wet with an annual rainfall of 3725 mm (mean from 1999 to 2010, Munda Airport, New Georgia Island in the Western Province and 2837 mm in the Central Province; Solomon Islands Bureau of Meteorology, unpublished data). Sixty-eight per cent of study village residents self-reported sleeping under a long-lasting insecticide treated net (unpublished data). In both provinces, the mean daily minimum and maximum temperatures were 24 °C and 30 °C, respectively, with an overall mean of 26 °C.

### Study period

Unless otherwise specified, anopheline biting densities were estimated in the Western Province between March 2014 and August 2016 for 4 nights per village survey (*n* = 2064 man-nights), and in Haleta (Central Province) between August 2011 and August 2016 for 5 nights per survey (*n* = 1534 man-nights). Entomological data reported previously for Haleta in Central Province [10, 14–16] is updated here for comparative analyses of vector species composition and heterogeneity in biting rates among and within villages.

### Sampling of adult anophelines

Host-seeking (biting) females were sampled with human landing catches (HLC) conducted from 18:00 to 00:00 h by village collectors working outdoors at 10 sites distributed throughout each village. To determine the all-night biting profile, mosquito collections were extended to 06:00 h in Jack Harbour and Saeragi during September 2014. Anophelines landing on the exposed legs and feet of collectors were captured by mouth aspiration and held in individual containers by hour and collection site. Anophelines were identified by morphological criteria [19], prior to dissection for parity determination [20]. Specimens were preserved in 100% ethanol for subsequent PCR-based identification using a DNA sequence of the internal transcribed spacer region 2 of the ribosomal DNA (ITS2) [21] and detection of *Plasmodium* DNA in heads and thoraces by nested PCR [22].

The annual entomological inoculation rate (EIR) was calculated from the product of the sporozoite rate and the annual biting rate [23, 24]. The sporozoite rate was defined as the proportion of mosquitoes with malaria specific DNA in the head or thorax. The all-night biting rate was calculated by adjusting the estimated biting rate from 18:00–24:00 h to account for the proportion of females estimated to have fed after midnight; based on the all-night collections conducted for *An. farauti* in Jack Harbour and *An. lungae* in Saraegi village.

### Statistical analysis

Data detailing mosquito surveys and their analyses by dissections and molecular analysis are available from the James Cook University Tropical Data Hub [25, 26]. Differences in the anopheline community composition among villages were analysed by permutational multivariate ANOVA (PERMANOVA; package *vegan*) [27] and displayed graphically using non-metric multidimensional scaling (*nMDS*) [28]. Temporal and spatial changes in each species biting rates were compared with a generalized linear model (GLM; package = *MASS*) with a negative binomial distribution and interacting fixed factors for sample period and village. All analyses were conducted using the R package V3.1.2 [29].

Geographical data were projected in ArcGIS (v10.0) [30] and local spatial clusters of high mosquito densities were detected using FleXScan (v3.1.2) [31] which can identify either circular or irregular shaped clusters [32]. The flexible scan statistic uses a purely spatial Poisson distribution model to identify spatially aggregated clusters with higher than average mosquito densities (“vector foci”) by identifying the spatial window with the greatest ratio of observed to expected cases (Relative Risk). Cluster detection was based on a spatial matrix [33] defined using triangular irregular networks created based on Delaunay Triangulation, with Euclidian distance.

## Results

**Fig. 2:**
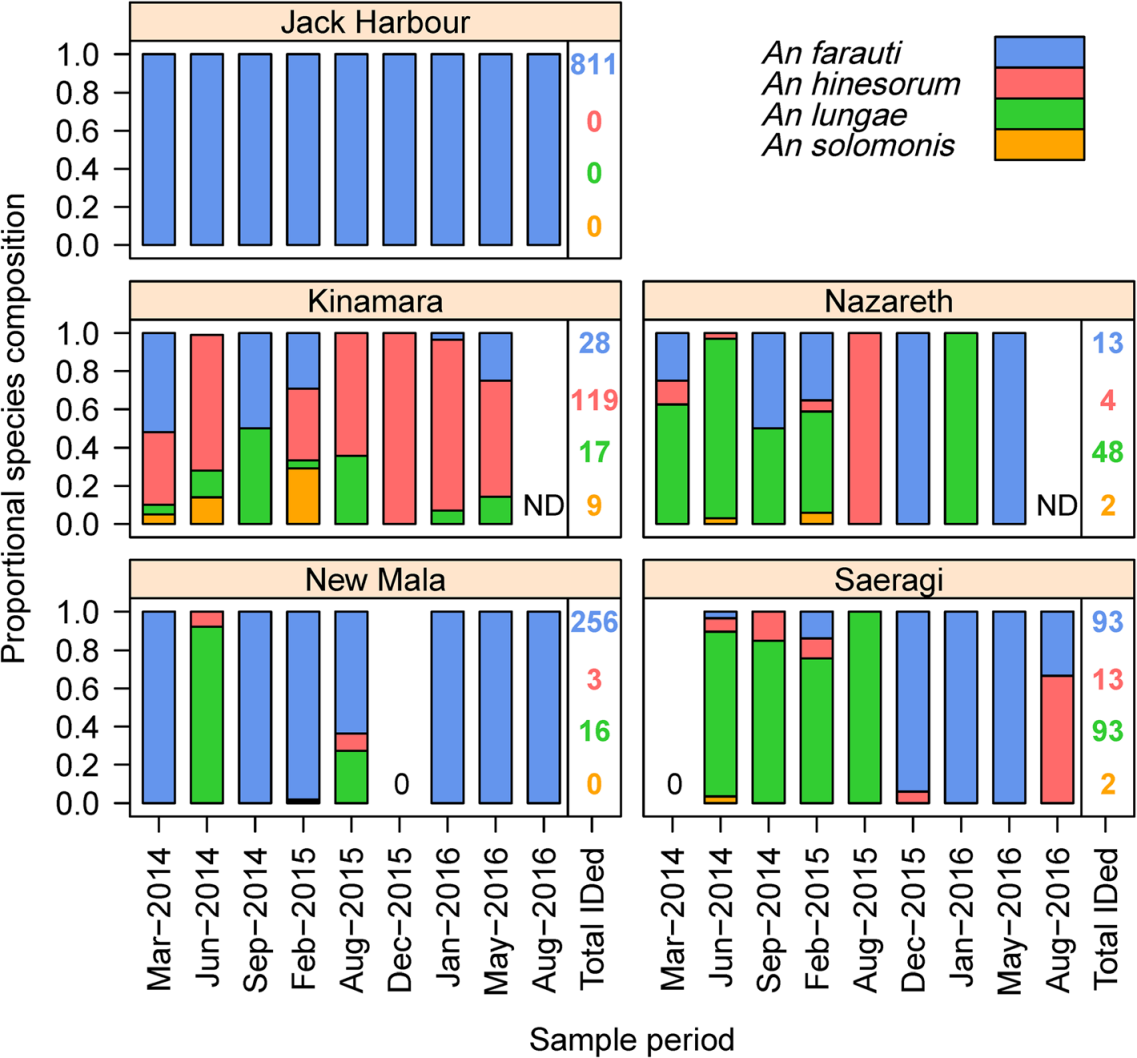
Temporal species composition of *Anopheles farauti*, *Anopheles hinesorum*, *Anopheles lungae* and *Anopheles solomonis* from villages in Western Province. Total numbers of *An. farauti*, *An. hinesorum*, *An. lungae* and *An. solomonis* identified by PCR are shown in blue, red, green and orange, respectively. Analyses of the Dec 2014 collection in Jack Harbour confirmed all anophelines were *An. farauti* (*n* = 96). Analyses across all collections in Obobulu confirmed 86 % *An. farauti* (*n* = 19) and 14 % *An. hinesorum* (*n* = 3). Analyses across all collections in Tuguivili confirmed 91 % *An. farauti* (*n* = 60), 3 % *An. hinesorum* (*n* = 2) and 6 % *An. lungae* (*n* = 4). *Key*: ND, no data; 0, no specimens were caught

**Fig. 3:**
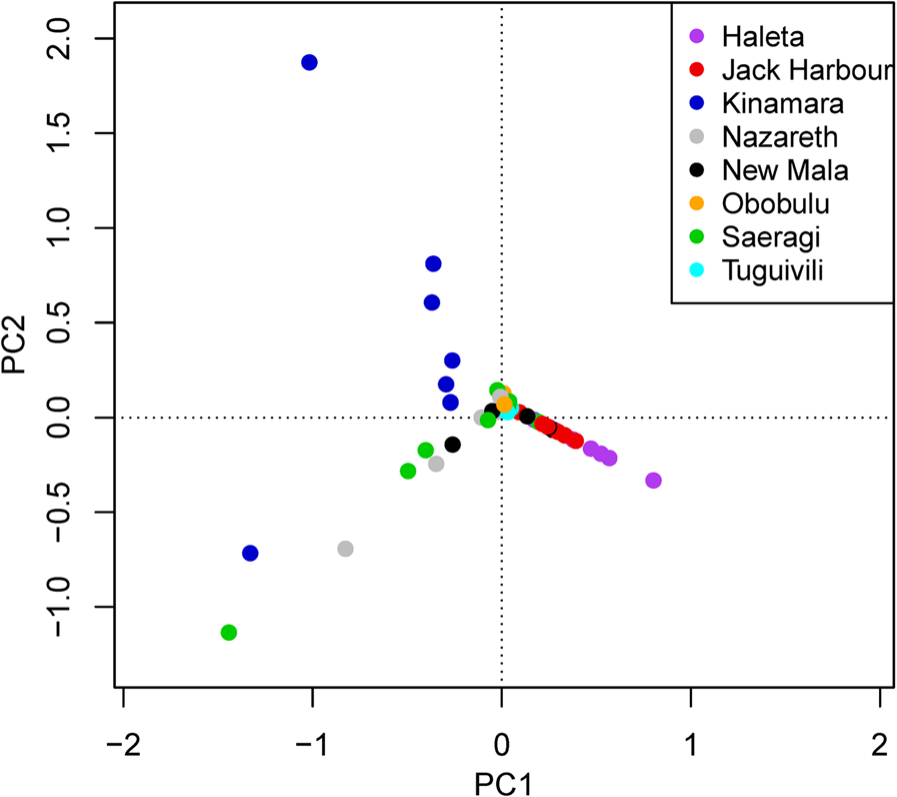
Non-metric multidimensional scaling ordination plot comparing the species abundance from different sample periods in study villages in Western (Jack Harbour, Kinamara, Nazareth, Obobulu, Saeragi) and Central (Haleta) Provinces. Each point represents the species composition of one village at one sampling period, and those that are more similar to one another are ordinated closer together. The axis and orientation of the plot is arbitrary

In the Western Province, mosquito surveys were conducted longitudinally in Jack Harbour (*n* = 10), New Mala (*n* = 9), Saeragi (*n* = 9), Kinamara (*n* = 8), Nazareth (*n* = 8), Obobulu (*n* = 5) and Tuguivili (*n* = 5) and once each in Boboe, Iriri, Kuzi and Koriovuku for a total of 2064 man-nights (each survey consisted of 4 nights of collections with 10 collectors per night; Table 1). Members of both the *An. farauti* (*s.l.*) (*n* = 11,516) and *An. lungae* (*s.l.*) (*n* = 187) complexes were collected outdoors with HLC. PCR analyses estimated that of those morphologically identified to belong to the *An. farauti* complex; 91% were *An. farauti* (1376/1520) and 9% *An. hinesorum* (144/1520; Fig. 2). Of the *An. lungae* complex specimens, 93% were confirmed by PCR as being *An. lungae* (178/191) and 7% were *An. solomonis* (13/191; Fig. 2). The species composition was significantly different among villages, both as a main effect (PERMANOVA, *F*_(1,75)_ = 2.37, *P* = 0.001) and as an interaction with time (*F*_(1,75)_ = 0.88, *P* = 0.011). Although the species composition was stable over time in some villages, e.g. Jack Harbour, there was strong temporal variation in others, e.g. Kinamara (Fig. 2). The anopheline communities ranged from being exclusively *An. farauti* in Jack Harbour (and Haleta in the Central Province) to a dominance of *An. lungae* in Nazareth and Saeragi with other villages (Kinamara) co-dominated by two species. In the ordination plot, the only village that consistently separated on a different gradient was Kinamara, which had varying mixed populations including proportionally elevated numbers of *An. hinesorum* and *An. solomonis* (Fig. 3).

**Table 1:** Timeline of anopheline surveys in Western Province, Solomon Islands

Island	Village^a^	Sample period									
		2014				2015			2016		
		Mar	Jun	Sep	Dec	Feb	Aug	Dec	Jan	May	Aug
Kolombangara	Jack Harbour	×	×	×	×	×	×	×	×	×	×
Gizo Island	Saeragi	×	×	×		×	×	×	×	×	×
Kohinggo	Kinamara	×	×	×		×	×	×	×	×	
	Nazareth	×	×	×		×	×	×	×	×	
	New Mala	×	×	×		×	×	×	×	×	×
Ranonnga	Obobulu	×	×	×		×	×				
New Georgia	Tuguivili						×	×	×	×	×

^a^All villages could not be sampled simultaneously and some surveys were conducted in the preceding or following month to the one indicated. Boboe (Kohinggo Island), Iriri and Kuzi (Kolombangara Island) and Koriovuku (Ranonnga Island) villages (not shown above) were each sampled at single time points

**Fig. 4:**
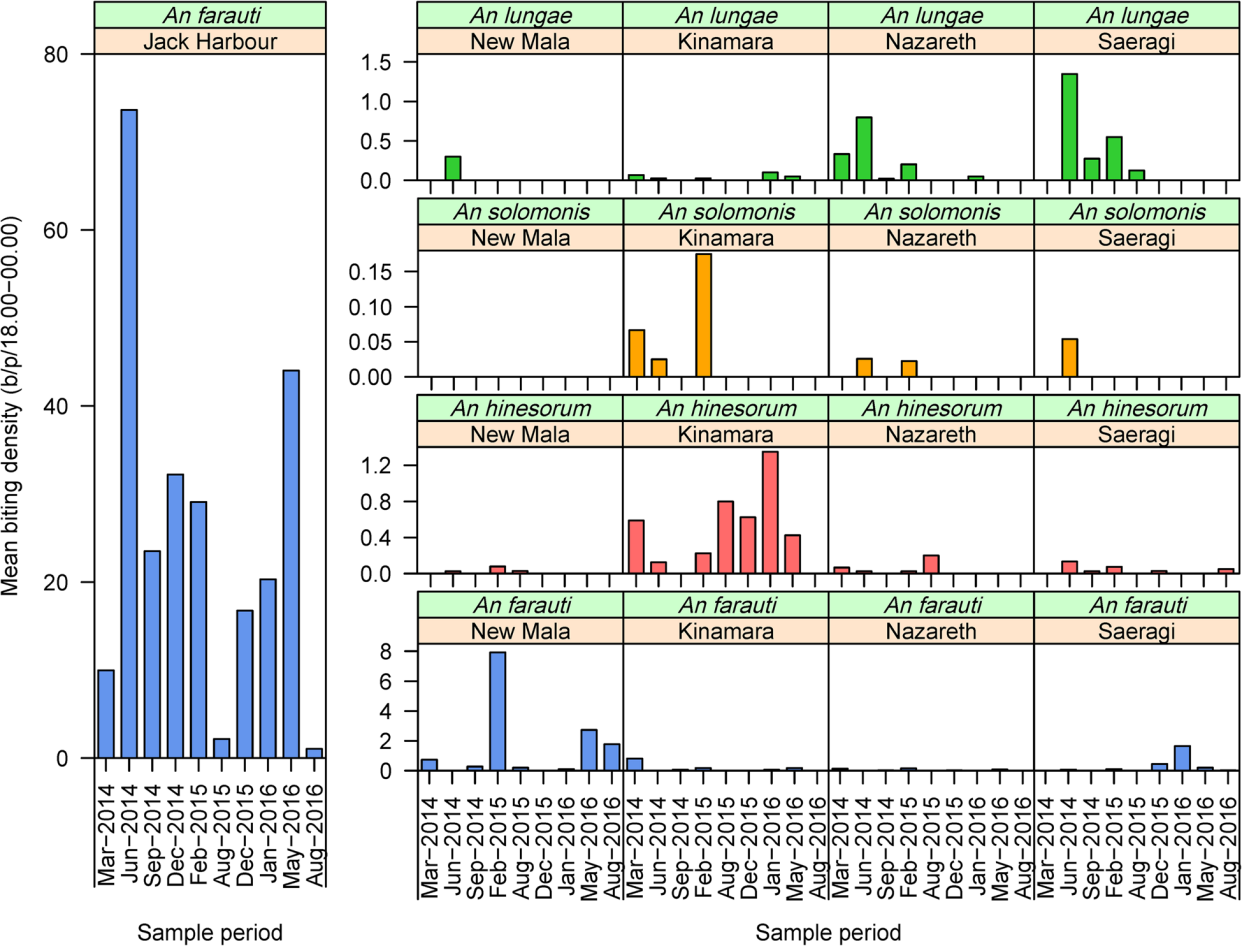
Longitudinal densities of *Anopheles farauti*, *Anopheles hinesorum*, *Anopheles lungae* and *Anopheles solomonis* in Jack Harbour, Kinamara, Nazareth, New Mala and Saeragi villages, Western Province, Solomon Islands, estimated by human landing catches from 18:00–00:00 h. Y-axes scale varies by anopheline species

With the exception of Jack Harbour where 10,530 *An. farauti* were captured by HLC, *An. farauti*, *An. hinesorum*, *An. lungae* and *An. solomonis* populations were not abundant in the other villages (Fig. 4). *Anopheles farauti* biting densities from 18:00 to 24:00 h were estimated as 26.3 bites per person per half-night (b/p/h-n) in Jack Harbour and 1.5 b/p/h-n in New Mala, with an average of 0.2 b/p/h-n in all other villages surveyed ≥ 8 times (Kinamara, Nazareth and Saeragi). Large heterogeneities in biting rates were observed for *An. farauti* over time (*β* = -0.303, *SE* = 0.072, *P* < 0.0001) and among villages (*β* = -0.982, *SE* = 0.075, *P* < 0.0001), noting that there was a significant interaction between time and village (*β* = 0.063, *SE* = 0.011, *P* < 0.0001). For example, the temporal variability of the average biting rate in Jack Harbour across the 10 collection periods ranged from a low of 1.2 b/p/h-n in August 2016 to a high of 73.7 b/p/h-n in June 2014. While the average biting rate of *An. hinesorum*, *An. lungae* and *An. solomonis* among all villages was always very low at 0.1, 0.1 and 0.008 b/p/h-n, respectively, although at times significant increases in the biting rates were observed for *An. hinesorum* (*β* = -0.414, *SE* = 0.162, *P* = 0108) and *An. lungae* (*β* = 0.489, *SE* = 0.209, *P* = 0.0194). However, *An. solomonis* densities remained consistently low (*β* = -0.112, *SE* = 0.954, *P* = 0.906). At such low densities, the GLM model did not detect any influence of village on the landing rates of *An. hinesorum* (*β* = -0.225, *SE* = 0.190, *P* = 0.237) or *An. solomonis* (*β* = -0.471, *SE* = 1.274, *P* = 0.711), whereas the densities of *An. lungae* were influenced by village (*β* = 1.014, *SE* = 0.155, *P* < 0.0001) with a significant interaction with time (*β* = -0.116, *SE* = 0.026, *P* < 0.0001). Seasonal patterns for any of the species or villages were not obvious (due to low densities), except for *An. farauti* in Jack Harbour, where a peak biting season occurred between April and June.

Five entomological surveys were made in Obobulu (March 2014 to August 2015) and Tuguivili (August 2015 to August 2016). In Obobulu, the mean density of *An. farauti* was 0.12 b/p/h-n and for *An. hinesorum* was 0.02 b/p/h-n, with neither *An. lungae* nor *An. solomonis* captured. In Tuguivili, the mean density of *An. farauti* was 0.56 b/p/h-n, for *An. hinesorum* was 0.03 b/p/h-n and for *An. lungae* was 0.006 b/p/h-n without any *An. solomonis* caught. Additional single entomological surveys were made in Koriovuku (June 2014), Iriri (October 2014), Kuzi (December 2014) and Boboe (July 2015) villages. In Korivuku, Iriri and Boboe neither *An. farauti* (*s.l.*) nor *An. lungae* (*s.l.*) were captured from 18:00 to 24:00 h. In Kuzi only *An. farauti* (*s.l.*) was collected (1.7 b/p/h-n).

The heads and thoraces of *An. farauti* (*n* = 1921), *An. hinesorum* (*n* = 39), *An. lungae* (*n* = 149) and *An. solomonis* (*n* = 13) were analysed by PCR for *Plasmodium* DNA as an indicator of malaria sporozoites in the salivary glands (Table 2). Hereafter, PCR positives from heads and thoraces will be referred to as sporozoite DNA. *Plasmodium falciparum* sporozoite DNA positive *An. farauti* were identified in Jack Harbour (*n* = 10) and New Mala (*n* = 6). Sporozoites of *P. vivax* were identified in *An. farauti* in Jack Harbour (*n* = 3) and New Mala (*n* = 2) with a single sporozoite positive *P. ovale* detected in *An. farauti* in Jack Harbour. The head and thorax of one *An. hinesorum* from Kinamara was positive for *P. falciparum* DNA. The overall sporozoite rate for *An. farauti* was 1.1% (*n* = 22/1921) and for *An. hinesorum* was 2.6% (*n* = 1/39; Table 2). *Plasmodium* DNA was not detected in *An. lungae* (*n* = 149) or *An. solomonis* (*n* = 13). For *An. farauti*, the overall annual EIR was estimated to be 26.6 infective bites/person/year (ib/p/y; Table 3) and ranged from 16.5 in 2014 to 55.5 in 2015. For *An. hinesorum*, the overall estimated EIR was 1.0.

**Table 2:** Sporozoite-positive *Anopheles farauti* and *Anopheles hinesorum* by village in the Western Province

Species	No. tested	Sporozoite positives (%)			
Village		*P. falciparum*	*P. vivax*	*P. ovale*	Total
*An. farauti* (*s.s.*)					
Jack Harbour	1756	0.57	0.17	0.06	0.8
Kinamara	19	0.00	0.00	0.00	0
Nazareth	9	0.00	0.00	0.00	0
New Mala	115	5.22	1.74	0.00	6.9
Obobulu	16	0.00	0.00	0.00	0
Saeragi	6	0.00	0.00	0.00	0
Summary all villages	1921	0.83	0.26	0.05	1.1
*An. hinesorum*					
Kinamara	22	4.54	0.00	0.00	4.5
Nazareth	2	0.00	0.00	0.00	0
New Mala	2	0.00	0.00	0.00	0
Obobulu	3	0.00	0.00	0.00	0
Saeragi	10	0.00	0.00	0.00	0
Summary all villages	39	2.63	0.00	0.00	2.6

*Note*: Sporozoites were not detected in either *An. lungae* (*n* = 149) or *An. solomonis* (*n* = 13)

**Table 3:** Estimated malaria transmission rates by members of the *Anopheles farauti* complex in Western Province

Year	No. tested	Sporozoite positives (%)				All night-biting rate^a^	Annual EIR^b^
		*P. falciparum*	*P. vivax*	*P. ovale*	*Plasmodium spp.*		
2014	1546	0.26	0.13	0.06	0.45	16.89	27.91
2015	591	2.03	0.51	0	2.54	6.44	59.73
Overall	2137	0.75	0.23	0.05	1.03	10.81	40.63

^a^The value for the all night biting rate was calculated from the landing catches from 18:00 to 00:00 h and adjusted from biting profile which estimated that 59% of biting occurs before midnight

^b^EIR [infective bites per person per year (ib/p/y)] = Sporozoite rate × Biting rate (6pm-6am) × 365; where the sporozoite rate for all *Plasmodium* species is used

*Note*: The villages included in these calculations were those with ≥ 8 sample periods, being Jack Harbour, Kinamara, Nazareth, New Mala, Saeragi

**Fig. 5:**
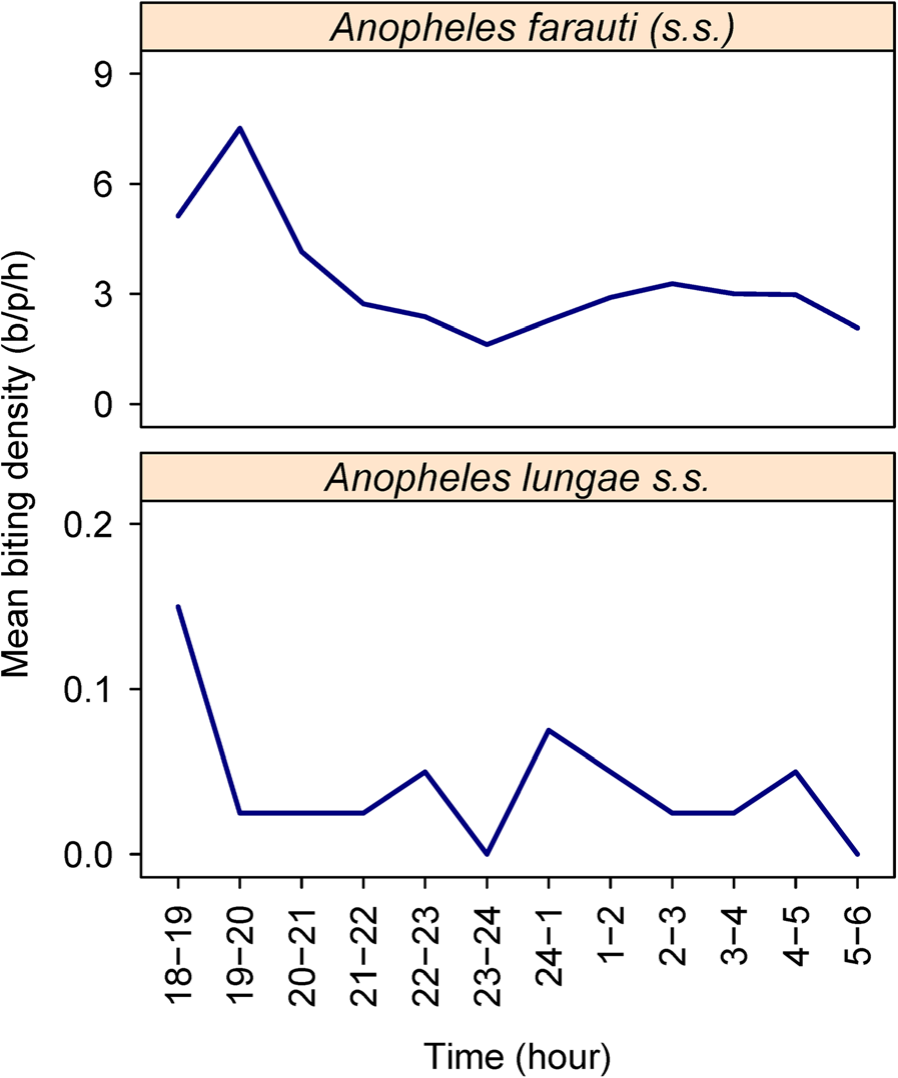
The hourly outdoor biting profiles of *An. farauti* in Jack Harbour village during September 2014 (top) and *An. lungae* in Saeragi village during June 2014 (bottom), Western Province, Solomon Islands

Peak biting of *An. farauti* occurred from 19:00 to 20:00 h (Fig. 5). The percentage of overall biting that occurred before 21:00 h was 49% and before midnight was 59%. For *An. lungae*, peak biting was even earlier (between 18:00–19:00 h) (Fig. 5). The percentage of overall biting by *An. lungae* that occurred before 21:00 h was 45% with 55% of biting before midnight.

Only Jack Harbour village had adequate numbers of *An. farauti* to allow multiple time-point parity rate estimations. The overall parity rate of *An. farauti* was 0.50 (393 parous of 785 dissected, pooling 6 surveys between May 2014 and August 2016). Parity varied significantly by time (*β* = -0.293, *SE* = 0.064, *P* < 0.0001), with parity estimates ranging between 0.33 and 0.70. Interestingly, the *An. farauti* parity rate in New Mala village was also 0.5 from 254 dissections across three survey periods.

**Fig. 6:**
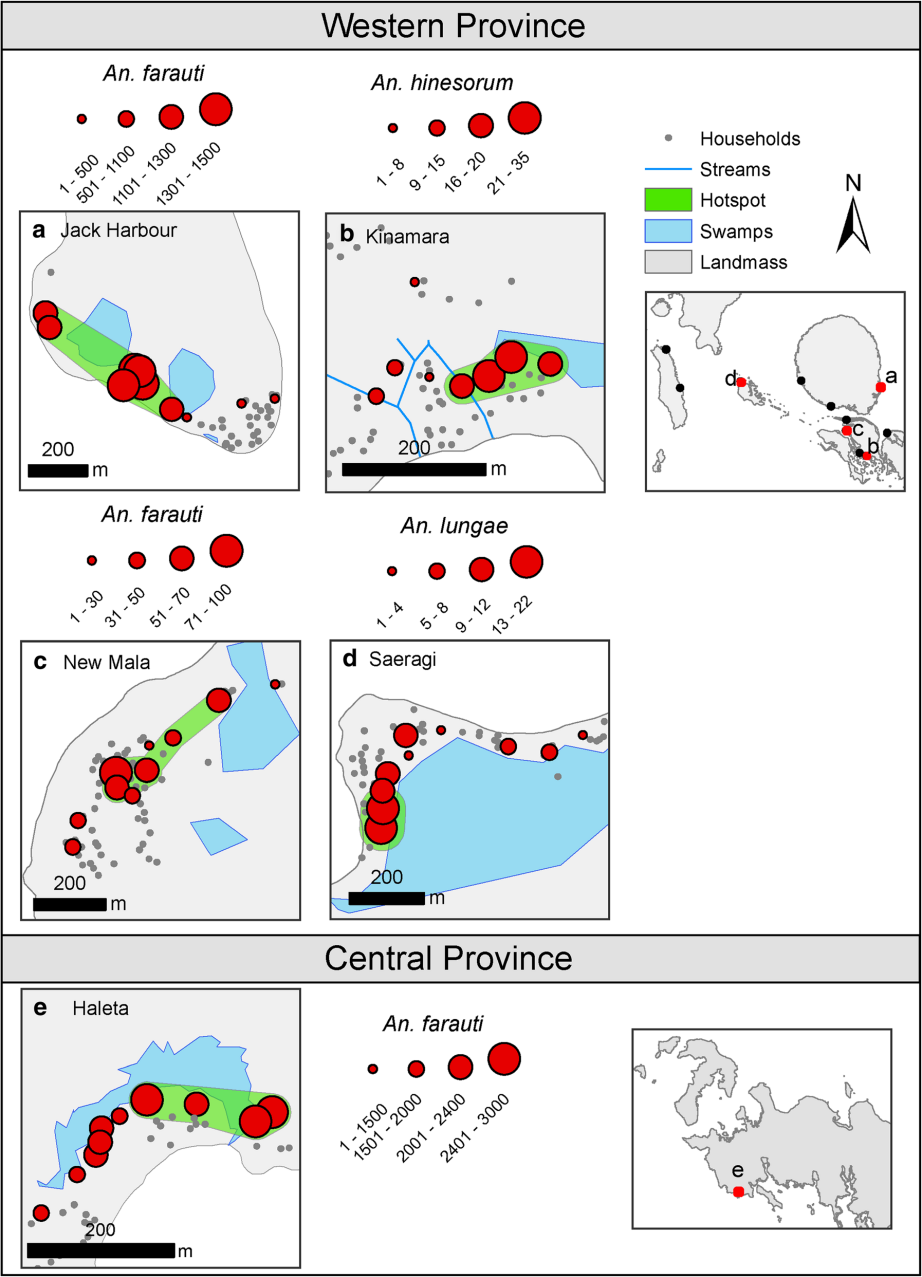
Spatial distribution and clustering of *An. farauti* densities in the Western Province villages of Jack Harbour and New Mala and the Central Province village of Haleta, as well as *An. hinesorum* in Kinamara village and *An. lungae* in Saeragi village. The panel labels (**a**-**e**) refer to the locations of each village on the regional maps. The scales differ by village and represent the total number of female mosquitoes caught in all sampling periods by HLC site

Small-scale spatial clustering (foci) of *An. farauti* densities was investigated in Jack Harbour, New Mala and Haleta; and for *An. hinesorum* in Kinamara and *An. lungae* in Saeragi. High spatial variations in biting rates within a village were observed during single surveys. In Jack Harbour village, for example, mean nightly biting catches among the 10 collection sites ranged from 1 to 129 b/p/h-n during the June 2014 survey. In each village, foci of higher than average densities of either *An. farauti*, *An. hinesorum* or *An. lungae* were identified (Fig. 6, Table 4). For *An. farauti*, the foci contained 4 to 7 sampling sites with the maximum distance across foci ranging from 170 m in Haleta to 558 m in Jack Harbour. Between 47% and 92% of anophelines were captured in foci. The *An. hinesorum* focus in Kinamara with a maximum size of only 126 m contained 4 sampling sites and accounted for 67% of all *An. hinesorum* captured. The *An. lungae* focus in Saeragi with a maximum size of only 52 m contained 2 sampling sites but accounted for 44% of all *An. lungae* captured.

**Table 4:** Spatial clusters (foci) of *An. farauti* densities within Jack Harbour, New Mala and Haleta villages as well as *An. hinesorum* in Kinamara village and *An. lungae* in Saeragi village

Village	Maximum distance (m)	Percent of locations (*n*/*N*)	Observed percent of mosquitoes (*n*/*N*)	Expected no. of mosquitoes	Relative risk (Obs/Exp)	*P*-value
*An. farauti* (*s.s.*)						
Jack Harbour	538	70 (7/10)	92 (9043/9870)	6909	1.31	0.001
New Mala	368	50 (5/10)	67 (337/500)	250	1.35	0.001
Haleta	170	40 (4/10)	47 (10,440/ 22,183)	8873	1.17	0.001
*An. hinesorum*						
Kinamara	126	40 (4/10)	67 (98/147)	58	1.67	0.001
*An. lungae* (*s.s.*)						
Saeragi	52	20 (2/10)	44 (39/95)	19	2.05	0.002

*Note*: Foci were detected with a flexible scan statistic using FleXScan software

## Discussion

Understanding vector species composition and abundance are important considerations for stratifying areas for targeting malaria vector control, particularly as transmission diminishes. The abundance of competent vector species in a suitable climate defines the relative receptivity of a strata for malaria transmission [34]. Receptivity coupled with an influx of parasites (which defines the malaria vulnerability) will guide malaria elimination programmatic decisions on when and where universal access to vector control can be reduced without increasing the risk of re-establishment of transmission after reintroduction of malaria parasites [11]. Malaria transmission in the Solomon Islands with one dominant vector provides a “simple” scenario to estimate transmission, transmission potential and receptivity and the underlying determinants. Anopheline human biting rates and sporozoite infections were analysed from longitudinal surveys among and within villages to define spatial and temporal heterogeneities associated with a malaria focus.

Four anopheline species (*An. farauti*, *An. hinesorum*, *An. lungae* and *An. nataliae*) were previously found during larval surveys in the Western Province from February to May 2013 [14]. While *An. farauti* was the predominant anopheline collected in HLCs, only 18% of larvae were identified as *An. farauti* from the seven villages in which both larval surveys and longitudinal monitoring of adult anophelines was conducted. *Anopheles hinesorum* was infrequently collected by HLC but was the most commonly collected larval species (66% of specimens) and was found in six of the seven villages, being only absent from Jack Harbour [14]. Particularly noteworthy was the anopheline species composition of Kinamara village in which *An. hinesorum* was the most frequent human biter (this species was previously reported as a non-human biter in the Solomon Islands [35, 36]). The ecology of Kinamara was unique among the study villages as multiple small fast-flowing steams are found within the village with thick emergent vegetation along the borders of these streams providing harbourage for *An. hinesorum* larvae.

In contrast to some previous studies, *An. lungae* and *An. hinesorum* were collected biting humans in the Solomon Islands, albeit in small numbers [10, 37, 38]. For *An. lungae*, this behaviour was widespread (though infrequent) in the Western Province, occurring in villages on Ranonngga, Vonavona, Gizho and Kolobangara islands. *Anopheles hinesorum* was infrequently collected by HLC in the villages of Kinamara, Nazareth, New Mala, Obobulu and Saeragi, despite being the most frequently collected anopheline in larval surveys [14]. *Anopheles solomonis* (which was not identified during larval surveys) was collected in limited numbers biting humans in the coastal villages of Kinamara, Nazareth and Saeragi. Human-biting *An. solomonis* was previously reported from an inland village on Santa Isabel Island [39]. The vector status of *An. lungae* and *An. solomonis* remains unresolved (sporozoites were not detected, but the sample sizes were inadequate for any definitive conclusions).

*Anopheles hinesorum* is a significant vector of *P. falciparum*, *P. vivax* and *P. malariae* in Papua New Guinea [40]. In the Solomon Islands, *An. hinesorum* was previously reported as an animal biter and therefore not a vector of human malarias [39] - until this study found *P. falciparum* DNA in the head and thorax of a single *An. hinesorum*, establishing this species as susceptible to infection with *P. falciparum*. However, its infrequent human-biting habit may limit its role in malaria transmission in the Solomon Islands.

Evidence for ongoing transmission of *P. falciparum*, *P. vivax* and *P. ovale* by *An. farauti* was found in the Western Province, a low malaria transmission/near elimination province. While 76% of human infections in the Western Province are *Plasmodium vivax* (unpublished data), *P. falciparum* sporozoites were detected more than 4-fold more frequently in *An. farauti* than *P. vivax* sporozoites. Furthermore, sporozoite rates across cross-sectional surveys varied widely (being frequently zero). Such disparities may be a consequence of low numbers of sporozoite- positive mosquitoes, sampling bias (including serendipitous placement of collection sites and timing of collection periods), measurement errors (including lack of precision and the inherent large fluctuations in mosquito population densities [41]), as well as the likely predominance of *P. vivax* infections due to relapses [42]. The difficulty and cost associated with both collecting an adequate number of anophelines of a given species, and their analyses for sporozoites makes the programmatic use of sporozoite rates impractical as a surveillance tool to monitor changes in transmission, particularly in low transmission scenarios. Similarly estimates of survivorship (by parity dissection) are often impractical due to the difficulty in capturing sufficient numbers of mosquitoes to track changes in survivorship in low transmission settings.

By extension, the entomological inoculation rate which is the product of the sporozoite rate and the biting rates would suffer from the same implementation constraints as the sporozoite rate in low transmission settings. Significant associations between EIRs and parasite prevalence in children were established for both *P. falciparum* and *P. vivax* in a high transmission area in Papua New Guinea [43] and across multiple sites in Africa for *P. falciparum* [23]. Analyses of EIRs in Africa questioned the use of the EIR as a means to estimate transmission as reductions in EIRs by 95% in some parts of Africa would be required before an impact on parasite rates in humans would be detectable [23]. Similarly, another study reported that the same human malaria prevalence was associated with a wide range of EIRs [44] while in other studies, high malaria rates (> 44%) had very low EIRs (< 0.001) [23].

Estimating the EIR is technically challenging, labor-intensive and costly, and gives only very imprecise estimates with low external validity [44–46]. In low transmission areas, EIR measurements as a surveillance tool are not feasible [44]. The discordant associations between infections in humans and EIRs may also be a function of the lack of standardized methods for estimating the EIR that includes not considering the ecological, demographic, and socioeconomic differences across populations [46].

Villages within (Jack Harbour) and near (New Mala) the high malaria focus had higher densities of the primary vector, *An. farauti* (26.3 and 1.5 b/p/h-n, respectively), than villages outside the foci (average of 0.2 in Kinamara, Nazareth and Saeragi) suggesting that in areas of low transmission intensity, landing rates may better serve as proxies for inoculation rates (David Smith, personal communication). However, estimating potential transmission intensity by vector landing rates is not without its own challenges as landing rates vary widely in time and space being influenced by numerous factors including weather patterns, larval habitats, adult survivorship and flight patterns as well as variations in the attractiveness to and efficiency of individuals to capture mosquitoes. Using the human landing rate (or other proxies for estimating biting rates, e.g. CDC light traps, double net traps, odour (human or animal) baited traps, as measures of receptivity and potential transmission intensity requires longitudinal and representative selection of multiple collection sites to adequately capture the heterogeneity in vector densities within and among villages and the seasonality that characterises vector populations and transmission in many areas. Such small-scale spatial variations and temporal heterogeneity in mosquito densities can have significant consequences for disease transmission [47] and their characterization needs to be adequately described to be programmatically useful [48].

The disparities documented between relative abundances of anopheline species between larval surveys and human biting rates in this study emphasizes the importance of understanding the behaviours of anophelines and their potential as malaria vectors. In this area, larval surveys were not reliable as predictors of relative receptivity for two reasons. Firstly, there is no known method to translate larval survey data to estimates of adult biting numbers. Secondly, a number of the anopheline species collected were infrequent human biters, especially *An. hinesorum*. Although *An. hinesorum* was the most commonly collected anopheline in larval surveys and although a single sporozoite-infected *An. hinesorum* was identified, this species was a very infrequent human biter and unlikely to be able to maintain malaria transmission.

## Conclusions

Despite the heterogeneity amongst and within villages, some common characteristics were found for vector bionomics in a malaria focus in the Solomon Islands. Villages within this malaria focus were consistent in that *An. farauti*, the dominant vector, was the most common anopheline present and was found in higher densities in villages within and near the foci compared to villages outside of the foci. Furthermore, the vector foci within villages in the malaria focus were larger, encompassing a greater proportion of the villages than villages outside the malaria focus. Thus, villages with both higher malaria receptivity and greater transmission were characterized by the presence of the dominant vector with consistently higher biting densities distributed over a larger area compared to villages of lower receptivity. As such, receptivity and potential transmission risk for programmatic decision-making may be most reliably estimated by the vector biting rate. In such low transmission settings, the other entomological indicators (sporozoite rates, entomological inoculation rates and parity rates) were difficult to estimate with precision due to low numbers of mosquitoes, sampling errors and biases.

## Abbreviations

b/p/h-n: bites/person/half-night
EIR: entomological inoculation rate
GLM: generalized linear model
GTS: Global Technical Strategy
HLC: human landing catch
ib/p/y: infective bites/person/year
IRS: indoor residual spraying
LLINs: long-lasting insecticidal nets
